# Identification of a Novel Strain of Human Papillomavirus from Children with Diarrhea in China

**DOI:** 10.1128/genomeA.00761-13

**Published:** 2013-10-03

**Authors:** Jie-mei Yu, Yuan-yun Ao, Guoyan Zhao, Li-li Li, David Wang, Zhao-jun Duan

**Affiliations:** National Institute for Viral Disease Control and Prevention, China CDC, Beijing, Chinaa; Department of Pathology and Immunology, Washington University School of Medicine, St. Louis, Missouri, USAb

## Abstract

A highly divergent human papillomavirus (HPV) strain, HPV-L55, was identified in fecal samples from children hospitalized with diarrhea in China. The L1 gene of HPV-L55 shares <75% identity with previously reported HPVs, indicating that this virus represents a novel type of HPV. Phylogenetic analysis classified this virus as a member of the gammapapillomaviruses.

## GENOME ANNOUNCEMENT

Human papillomaviruses (HPVs) are a diverse class of small, circular, double-stranded DNA viruses in the family *Papillomaviridae* (1). *Papillomaviridae* currently includes >155 types grouped into five genera: *Alphapapillomavirus*, *Betapapillomavirus*, *Gammapapillomavirus*, *Mupapillomavirus*, and *Nupapillomavirus* (2). Recent technological advancements have led to the identification of novel HPV species, including HPV-SD2 (3), HPV-159 (4), and HPV-Fin864 (5). Here, we describe a novel HPV type (HPV-L55) that was identified using 454 pyrosequencing of fecal samples from a 4-month-old child hospitalized with diarrhea.

Using the customized informatics pipeline VirusHunter (6, 7), we identified a unique 380-bp read, which closely aligns to the E6 region of HPV-48, showing 44.2% identity at the nucleotide (nt) level. Further analysis showed that this read shared the highest nucleotide identity (70%) with HPV-Fin864, whose complete sequence was recently deposited in GenBank (5).

The full HPV-L55 genome was sequenced using the genome walking method (GenomeWalker kit; TaKaRa, Japan). The primers were designed based on the original 454 pyrosequencing read, along with newly amplified HPV-L55 sequences; long fragments were further amplified using Ex Taq DNA polymerase (TaKaRa, Japan) for final sequence confirmation. The sequences were then aligned and manually edited to produce the complete viral genome assembly. The complete circular genome of HPV-L55 is 7,277 bp in length with a G+C content of 37.47%.

The HPV-L55 genome encodes seven open reading frames (ORFs) (E6, E7, E1, E2, E4, L2, and L1), of which the putative E4 ORF contains a start codon and completely overlaps the E2 ORF. The genome contains a nucleotide-binding helicase domain (GPSNTGKS [G-X4-GKT/S]) in the E1 protein, two conserved zinc-binding domains of CxxC(x)_29_CxxC separated by 36 amino acids in the E6 protein, and a modified zinc-binding domain CxC(x)_29_CxxC in the E7 protein (4, 5, 8, 9). In addition, the HPV-L55 genome encodes a 442-bp upstream regulatory region (URR), which contains a TATA box (TATAAA, nt positions 22 to 27) and a polyadenylation signal (AATAAA, nt positions 7126 to 7121).

The full-length genome sequence of HPV-L55 shares the highest similarity with that of HPV-Fin864, with 72% identity at the nt level and 74% identity in the amino acid sequence of the capsid protein (L1). HPVs have traditionally been classified into different types based on the criterion that each type exhibits <90% similarity to the L1 nt sequence of other HPVs (2). Using this criterion, the HPV-L55 strain identified in this study represents a novel type of HPV. By using the Basic Local Alignment Search Tool (BLAST), HPV-L55 aligned most closely with the genus *Gammapapillomavirus* of *Papillomaviridae*, which currently includes >10 viral species known to infect humans (2, 5).

Further studies will be necessary to determine the prevalence of this virus in the general population. Although a direct association between HPV-L55 and human disease has yet to be established, the full-genome sequence of HPV-L55 described here will be important for determining the full picture of genetic diversity in HPVs.

### Nucleotide sequence accession number.

The full HPV-L55 genome sequence was deposited in GenBank under the accession no. KF482069.
